# Discrimination in the United States: Experiences of Asian Americans

**DOI:** 10.1111/1475-6773.13225

**Published:** 2019-10-27

**Authors:** Caitlin L. McMurtry, Mary G. Findling, Logan S. Casey, Robert J. Blendon, John M. Benson, Justin M. Sayde, Carolyn Miller

**Affiliations:** ^1^ Department of Health Policy and Management Harvard T.H. Chan School of Public Health Boston Massachusetts; ^2^ Research, Evaluation, and Learning Unit Robert Wood Johnson Foundation Princeton New Jersey

**Keywords:** Asian Americans, discrimination, racial disparities in health and health care, racism, social determinants of health, survey research

## Abstract

**Objective:**

To examine experiences of racial discrimination among Asian Americans, which broadly contribute to poor health outcomes.

**Data Source and Study Design:**

Data come from a nationally representative, probability‐based telephone survey, including 500 Asian and a comparison group of 902 white US adults, conducted January to April 2017.

**Methods:**

We calculated the percent of Asian Americans reporting discrimination in several domains, including health care. We used logistic regression to compare the Asian‐white difference in odds of discrimination, and among Asians only to examine variation by geographic heritage group (South Asian versus East Asian) and gender.

**Principal Findings:**

13 percent of Asians reported discrimination in healthcare encounters. At least one in four adults reported experiencing discrimination in employment (27 percent job applications, 25 percent equal pay/promotions); housing (25 percent); and interpersonal interactions (35 percent microaggressions, 32 percent racial slurs). In unadjusted models, East and South Asians were more likely than whites to report experiences of institutional discrimination, and South Asians were more likely than whites to report microaggressions. In adjusted models, Asians had higher odds than whites of reporting avoiding health care due to discrimination concerns and also when obtaining housing.

**Conclusions:**

Asians in the United States experience discrimination interpersonally and across many institutional settings, including housing and health care. South Asians may be especially vulnerable to forms of institutional discrimination and microaggressions. These results illustrate a need for greater investigation into the unique experiences of Asian subgroups and greater protections for groups at higher risk of discrimination, within health care and beyond.

## 1 INTRODUCTION

Despite a legacy of exclusionary immigration laws,^1^, ^2^ periods of forced migration and internment,^3^ and multiple high‐profile hate crimes against Asians in recent years,^4^, ^5^ patterns of discrimination against Asian Americans remain understudied at the population level. This is partly due to the difficulty of sampling the small, numerous, and culturally distinct groups that comprise Asians in the United States.^6^, ^7^, ^8^ Nonetheless, few empirical studies in the United States have examined anti‐Asian, racially motivated discrimination in recent decades.

In order to identify appropriate policies or interventions to address ongoing racial discrimination against Asians in the United States, it is important to first systematically inventory the ways in which Asian Americans may experience discrimination across a broad spectrum of life domains. It is also important to examine the experiences of those who might be at greater risk of harm from discrimination, including Asians from ethnic groups targeted for hate crimes^9^ and Asian women, who are more likely to experience adverse health effects from lower levels of racial discrimination, compared to Asian men.^10^ This study offers a starting point for such examination, using an original and nationally representative sample.

A growing body of evidence documents the relationship between racial discrimination and negative physical, mental, and behavioral health outcomes among Asians in the United States.^11^, ^12^, ^13^, ^14^, ^15^, ^16^ For example, a 2009 review of the literature on discrimination and health among Asian Americans shows a consistent association between discrimination and increased risk of health problems, including negative mental health outcomes such as depressive disorders and increased substance use; worse physical functioning and cardiovascular health; and increased risk of diabetes, obesity, and high cholesterol.^11^ More recently, a meta‐analysis of studies of racial discrimination and mental health among Asian Americans revealed significant relationships between racial discrimination and depression and anxiety.^12^ Evidence also suggests that experiences of discrimination among Asians in the United States, including perceived discrimination and related health outcomes, may vary by sociodemographic characteristics such as age, education, income, and ethnicity.^17^, ^18^, ^19^, ^20^, ^21^, ^22^

Research shows that the experience of discrimination—whether through institutional barriers or interpersonal interactions (eg, racial slurs used by one individual against another)—impacts health by operating as an ongoing stressor, causing progressive wear and tear on the body, which ultimately results in worse health outcomes.^23^, ^24^, ^25^ Recent research also suggests that discrimination may affect health through less direct means. For example, perceived discrimination in healthcare settings may decrease the use of formal healthcare services among some Asian Americans, especially women.^26^, ^27^

Increasing evidence about the health risks of discrimination, along with historical institutionalized discrimination against Asians in the United States, suggests an updated examination across a broad range of areas is warranted. This study, alongside complementary articles in this issue of *Health Services Research*, brings a public health perspective to the complexity and pervasiveness of discrimination in the United States today. It was conducted as part of a larger survey fielded in 2017 in response to a growing national debate about discrimination in the United States to understand experiences of discrimination against multiple different groups in America today, including blacks, Latinos, Asian Americans, Native Americans, women, and LGBTQ adults.^28^

This study has three purposes. First, it aims to document the prevalence of racial discrimination against Asian Americans across multiple institutional and interpersonal domains raised as areas of concern among experts,^28^ including health care, education, employment, housing, political participation, police, the criminal justice system, slurs, microaggressions (negative assumptions or insensitive/offensive comments), racial fear, harassment, and violence. Second, it compares Asian American experiences of racial discrimination to those of whites in the United States to add to existing disparities research. Finally, it examines subgroups of Asian Americans, to examine variation in experiences of discrimination by self‐reported heritage and gender.

## 2 METHODS

### 2.1 Study design and sample

Data were obtained from an original, nationally representative, probability‐based telephone (cell and landline) survey of US adults, conducted from January 26 to April 9, 2017. The survey was jointly designed by Harvard TH Chan School of Public Health, the Robert Wood Johnson Foundation, and National Public Radio. An independent firm, SSRS, administered the survey. Because Harvard researchers were not directly involved in data collection and de‐identified datasets were used for analysis, the study was determined to be “not human subjects research” by the Harvard TH Chan School of Public Health Office of Human Research Administration.

The full sample included 3453 US adults aged 18 years and older, including nationally representative samples of African Americans, Latinos, Asian Americans, Native Americans, white Americans, men, women, and LGBTQ people. This paper examines the full subsamples of 500 Asian (non‐Hispanic) and 902 white (non‐Hispanic) US adults. Throughout the paper, we use shorthand descriptors of Asian or white. To identify Asian and white adults, screening questions regarding racial and ethnic identities were asked at the beginning of the survey. This method of screening also allowed interviewers to use the appropriate language in survey questions to describe or refer to the respondent's own identity. For example, this allowed questions to be read as “Did you experience [form of discrimination] because you are [‘Asian American’]?” rather than “because of your race or ethnicity?” If respondents identified as multiracial, interviewers asked respondents which race they identified with most, and any following questions about racial/ethnic‐identity were based on that self‐identification.

The completion rate for this survey was 74 percent among respondents who answered initial demographic screening questions, with a 10 percent overall response rate, calculated based on the American Association for Public Opinion Research's (AAPOR) RR3 formula.^29^ Because data from this study were drawn from a probability sample and used the best available sampling and weighting practices in polling methods (eg, 68 percent of interviews were conducted by cell phone, and 32 percent were conducted via landline), they are expected to provide accurate results consistent with surveys with higher response rates.^30^, ^31^, ^32^

### 2.2 Survey instrument

The poll asked about adults’ personal experiences of racial discrimination. We conceptualized racial/ethnic discrimination as differential or unfair treatment of individuals based on self‐identified race, whether by individuals (based on beliefs, words, and behavior) or social institutions (based on laws, policies, institutions, and related behavior of individuals who work in or control these laws, policies, or institutions).^33^, ^34^, ^35^ We analyzed 18 questions from the survey, covering six institutional and six interpersonal areas of discrimination (question wording in Appendix S1). Institutional areas included employment, education, health care, housing, political participation, police encounters, and treatment by the courts. Interpersonal areas included racial slurs, microaggressions, racial fear, sexual harassment, being threatened or nonsexually harassed, and violence. We also examined two areas in which concerns about discrimination might prevent adults from taking potentially needed action: seeking health services and protection from the police. Our survey asked about discrimination in domains previously demonstrated to be associated with health, as well as some that were not, in order to capture a wide range of possible discriminatory experiences across adults’ lives.

Questions about experiences were only asked among a random half sample of respondents to maximize the number of questions while limiting response burden. Additionally, questions were only asked of relevant subgroups (eg, college questions only asked among adults who had ever applied to college). Items on harassment, violence, and avoidance due to anticipated discrimination asked about the respondent and family members due to the sensitive nature of the topic and prior literature demonstrating that vicarious experiences of stress (eg, through discrimination experienced by family members) can adversely affect individuals.^36^

### 2.3 Statistical analyses

Table 2 displays results from our first set of analyses. We began by calculating the proportion of all Asian Americans and all whites who reported that they had ever experienced racial discrimination in each domain. Second, because Asian Americans are a diverse group, we disaggregated experiences of discrimination in each domain by heritage group to assess whether certain Asian subpopulations were more likely to experience racial discrimination than whites. To form these groups, Asian respondents were asked to self‐identify their or their family's heritage. If respondents included more than one ethnicity, they were asked with which they identify more. Since our sample size precluded analyses of each ethnicity or nationality separately, these responses were divided into three broad geographic groupings. The heritage group “East Asians” includes respondents who reported Chinese, Japanese, Korean, or Taiwanese heritage (n = 232). “South Asians” include respondents who reported Indian, Bangladeshi, Pakistani, or Sri Lankan heritage (n = 156). Finally, “Southeast Asians” include respondents who reported Filipino, Vietnamese, Cambodian, Indonesian, Laotian, or Malaysian ancestry (n = 87). Using pairwise t tests of differences in proportions, we made uncontrolled comparisons of the percentage of whites and Asians—overall and in each heritage group—reporting racial discrimination. To keep our Type 1 error rate at 0.05, we used the Holm‐Bonferroni method to sequentially correct for multiple comparisons between groups and between models. It adjusts the threshold of statistical significance for each hypothesis test based on the rank order of its *P*‐value and the total number of tests conducted. We discuss only differences that achieve statistical significance using this approach in the results.

We then used logistic regression models to assess whether reporting discrimination remained significantly associated with race between Asians and whites after controlling for the following variables related to experiences of discrimination: gender (male, female), age (continuous), household income (<$50 000, $50 000+), education (less than college degree or at least a college graduate), current health insurance status (for healthcare outcomes only: insured or uninsured), neighborhood racial composition (measured as whether or not respondents live in a neighborhood they describe as predominantly their own race), metropolitan status (urban, suburban, rural), and region (US Census Bureau 4‐region division: Midwest, Northeast, South, and West).

Finally, we estimated logistic regression models exclusively among Asian adults to examine variation in experiences of institutional discrimination by heritage group (East Asians compared to South Asians) and gender, while controlling for household income (<$50 000/year or $50 000+/year), education (less than college degree or at least a college graduate), and age (continuous). Southeast Asians were excluded from this analysis due to small sample size. Modeling choices, especially as they concern dichotomous variable thresholds, were driven by sample size limitations. Models only included the subset of respondents who were asked each question. Due to small sample size, we were not able to test interactions in any models. To test the sensitivity of these results to different model specifications, we fit alternate models using different measures of income (household income less/greater than $75 000 per year), education (less than college degree, college degree, or postgraduate degree; less/greater than postgraduate degree), and age (less/greater than 30, 40, and 50 years old). We also dropped covariates one‐by‐one to examine whether our statistically significant results held. For Table 3, we tested the inclusion of a variable measuring whether the respondent was born within or outside of the United States. Results of these robustness checks are discussed below.

To compensate for known biases in telephone surveys (eg, nonresponse bias) and variations in probability of selection within and across households, sample data were weighted by household size and composition, cell phone/landline use, and demographics (gender, age, education, race/ethnicity, and census region) to reflect the true population distribution of Asian and white adults in the country. Other techniques, including random‐digit dialing, replicate subsamples, and random selection of a respondent within a household, were used to ensure that the sample is representative. All analyses were conducted using STATA version 15.0 (StataCorp) and all tests accounted for the variance introduced by weighted data.

## 3 RESULTS

### 3.1 Characteristics of the Asian study sample

Weighted characteristics of US Asians and whites in this study sample are presented in Table 1, overall and by Asian heritage group. Asian Americans differed from whites on many demographic measures, including age (37 percent of Asians were ages 50+, compared to 52 percent of whites, *P* < .001) and education (54 percent of Asians had a college degree or more, compared to 34 percent of whites, *P* < .001). Asians also differed from whites on three measures related to residence. Asian adults were less likely than whites to report living in a neighborhood comprised predominantly of people their own race (20 percent of Asians reporting living in predominantly Asian neighborhoods, compared to 67 percent of whites living in predominantly white neighborhoods) (*P* < .001). Asians were also more likely than whites to live in suburban areas (66 percent vs 53 percent, *P* < .001), less likely to live in rural areas (5 percent vs 25 percent, *P* < .001), and more likely to live in the Western United States. (45 percent vs 18 percent, *P* < .001). About one‐quarter (26 percent) of Asians in this sample were born in the United States.

**Table 1:** Characteristics of a nationally representative sample of Asian adults in the United States, overall and by geographic heritage groups

	Race/ethnicity		Geographic heritage groups		
	Whites^a^ (N = 902)	All Asians^b^ (N = 500)	East Asian^c^ (N = 232)	South Asian^d^ (N = 156)	Southeast Asian^e^ (N = 87)
	Percentage of respondents^f^				
Gender^g^					
Male	48	50 (*P* = .645)	43 (*P* = .326)	63 (*P* = .015)	43 (*P* = .481)
Female	52	50 (*P* = .645)	57 (*P* = .326)	37 (*P* = .015)	57 (*P* = .481)
Age (y)					
18‐29	18	24 (*P* = .070)	24 (*P* = .129)	18 (*P* = .943)	28 (*P* = .092)
30‐49	30	**39** k (*P* = .016)	30 (*P* = .903)	**57** k (*P* < .001)	24 (*P* = .351)
50‐64	29	22 (*P* = .046)	24 (*P* = .266)	21 (*P* = .111)	25 (*P* = .474)
65+	23	15 (*P* = .008)	22 (*P* = .760)	**3** k (*P* < .001)	23 (*P* = .980)
Education					
No college degree^h^	66	**46** k (*P* < .001)	54 (*P* = .013)	**20** k (*P* < .001)	64 (*P* = .836)
College degree or more	34	**54** k (*P* < .001)	46 (*P* = .015)	**80** k (*P* < .001)	36 (*P* = .799)
Household income					
<$25 000	23	24 (*P* = .636)	30 (*P* = .138)	15 (*P* = .076)	24 (*P* = .878)
$25 000‐<$50 000	22	13 (*P* = .004)	14 (*P* = .032)	**8** k (*P* < .001)	16 (*P* = .306)
$50 000‐<$75 000	11	12 (*P* = .797)	12 (*P* = .779)	10 (*P* = .673)	18 (*P* = .227)
$75 000+	35	41 (*P* = .133)	31 (*P* = .304)	**62** k (*P* < .001)	34 (*P* = .824)
Health insurance current status					
Uninsured	9	11 (*P* = .498)	12 (*P* = .484)	6 (*P* = .247)	14 (*P* = .327)
Insured, Medicaid primary source	6	5 (*P* = .918)	6 (*P* = .756)	3 (*P* = .311)	5 (*P* = .791)
Insured, non‐Medicaid primary source	84	83 (*P* = .733)	81 (*P* = .483)	91 (*P* = .084)	81 (*P* = .554)
Living in a neighborhood that is predominantly own race/ethnicity^i^	67	**20** k (*P* < .001)	**26** k (*P* < .001)	**17** k (*P* < .001)	**15** k (*P* < .001)
Area of residence					
Urban	17	23 (*P* = .069)	23 (*P* = .175)	21 (*P* = .335)	24 (*P* = .255)
Suburban	53	**66** k (*P* < .001)	67 (*P* = .005)	**74** k (*P* < .001)	58 (*P* = .478)
Rural	25	**5** k (*P* < .001)	**3** k (*P* < .001)	**0.3** k (*P* < .001)	14 (*P* = .048)
US region of residence^j^					
Northeast	18	20 (*P* = .492)	22 (*P* = .411)	25 (*P* = .160)	12 (*P* = .191)
Midwest	25	**10** k (*P* < .001)	**4** k (*P* < .001)	13 (*P* = .011)	11 (*P* = .003)
South	35	**19** k (*P* < .001)	**13** k (*P* < .001)	25 (*P* = .037)	26 (*P* = .118)
West	18	**45** k (*P* < .001)	**53** k (*P* < .001)	32 (*P* = .011)	**49** k (*P* < .001)
Born in the United States	—	26	33	**13** l (*P* < .001)	32

a Non‐Hispanic white adults aged 18+.

b Non‐Hispanic Asian adults aged 18+.

c East Asians include adults who report being of Chinese, Japanese, Korean, and Taiwanese heritage.

d South Asians include adults who report being of Indian, Bangladeshi, Pakistani, and Sri Lankan heritage.

e Southeast Asians include adults who report being of Filipino, Vietnamese, Cambodian, Indonesian, Laotian, and Malaysian heritage.

f Percentages may not add up to 100% due to rounding and don't know/refused responses that are included in the total n but not reported in this table.

g Gender indicates self‐ or interviewer‐reported gender of respondent.

h Includes those with some college experience (including business, technical, or vocational school after high school) but no college degree, as well as those with a high school degree or GED certificate or less.

i Question asked as: “People often describe some neighborhoods or areas as predominantly one group or another, such as a predominantly black or white neighborhood. Would you say that the area where you live is predominantly [Asian American OR white], or not?”

j Regions defined by US Census Bureau 4‐region definition.

k Bivariate comparisons significantly different from whites at *α* = 0.05 using the Holm‐Bonferroni method to sequentially correct for 108 comparisons between groups and models (shown in bold).

l Pairwise comparisons between geographic heritage groups significantly different from each other using the Holm‐Bonferroni method to correct for multiple comparisons between groups (shown in bold).

Asian adults also differed from whites on several sociodemographic characteristics when examined by geographic heritage groups (Table 1). For example, South Asians tended to be younger than whites (75 percent vs 48 percent under age 50, *P* < .001), more educated than whites (80 percent of South Asians had a college degree or more, compared to 34 percent of whites, *P* < .001), were more likely to live in the suburbs (74 percent vs 53 percent, *P* < .001), and had higher annual household incomes (62 percent of South Asians had incomes of $75 000+, compared to 35 percent of whites, *P* < .001). All three Asian heritage groups were less likely than whites to live in a neighborhood that is predominantly comprised of people from their own race or ethnicity (26 percent of East Asians, 17 percent of South Asians, and 15 percent of Southeast Asians, compared to 67 percent of whites, *P* < .001). Also, East Asians were less likely than whites to live in the Midwest (4 percent vs 25 percent, *P* < .001) or South (13 percent vs 35 percent, *P* < .001).

### 3.2 Reported experiences of racial discrimination overall

Table 2 shows that sizeable shares of Asian American adults reported having personally experienced institutional and interpersonal discrimination in several domains. In the context of institutional discrimination, more than a third (37 percent) of Asian adults say they have experienced racial discrimination in one or more domains. One‐quarter or more of Asian adults reported personally experiencing discrimination in employment (27 percent when applying to jobs, 25 percent in obtaining equal pay/promotions) and housing (25 percent). About one in six Asian adults reported experiencing discrimination when applying to or while attending college (19 percent) and when interacting with police (18 percent). More than one in 10 Asians (13 percent) reported discrimination when going to a doctor or health clinic, while 7 percent reported discrimination when trying to vote or participate in politics.

**Table 2:** Unadjusted differences between white and Asian adults in reporting discrimination because of race, overall and by geographic heritage groups

	Subject of discrimination^a^	N^d^	Race/ethnicity		Geographic heritage groups		
			Whites^b^	All Asians^c^	East Asian^e^	South Asian^f^	Southeast Asian^g^
*Belief in overall discrimination*							
General belief that discrimination against [your race] exists today in the United States^h^	All adults	1402	55	61 (*P* = .160)	62 (*P* = .175)	65 (*P* = .088)	56 (*P* = .898)
*Personal experiences of institutional discrimination*							
Ever experienced institutional discrimination	You or family member	1402	22	**37** n (*P* < .001)	**39** n (*P* < .001)	**46** n (*P* < .001)	25 (*P* = .658)
Employment							
Being paid equally or considered for promotions^i^	You	619	13	25 (*P* = .012)	24 (*P* = .097)	34 (*P* = .014)	13 (*P* = .940)
Applying for jobs^j^	You	621	19	27 (*P* = .120)	27 (*P* = .195	34 (*P* = .066)	13 (*P* = .364)
Education							
Applying to or while attending college^k^	You	612	11	19 (*P* = .055)	25 (*P* = .061)	15 (*P* = .433)	20 (*P* = .261)
Health care							
Going to a doctor or health clinic	You	745	5	13 (*P* = .011)	11 (*P* = .218)	17 (*P* = .058)	15 (*P* = .092)
Housing							
Trying to rent a room/apartment or buy a house^l^	You	624	5	**25** n (*P* < .001)	24 (*P* = .009)	24 (*P* = .012)	30 (*P* = .013)
Political participation							
Trying to vote or participate in politics	You	657	4	7 (*P* = .221)	5 (*P* = .692)	13 (*P* = .137)	7 (*P* = .592)
Police and courts							
Interacting with police	You	657	10	18 (*P* = .041)	15 (*P* = .265)	25 (*P* = .049)	17 (*P* = .407)
Unfairly stopped or treated by the police	You or family member	657	6	12 (*P* = .116)	5 (*P* = .676)	25 (*P* = .017)	7 (*P* = .934)
Unfairly treated by the courts	You or family member	657	7	6 (*P* = .715)	4 (*P* = .394)	7 (*P* = .997)	5 (*P* = .648)
*Personal experiences of interpersonal discrimination* m							
Ever experienced interpersonal discrimination	You or family member	1402	27	35 (*P* = .014)	36 (*P* = .038)	41 (*P* = .014)	28 (*P* = .786)
Microaggressions	You	745	19	35 (*P* < .001)	33 (*P* = .030)	**45** n (*P* < .001)	24 (*P* = .540)
Racial slurs	You	745	23	32 (*P* = .053)	36 (*P* = .064)	36 (*P* = .083)	25 (*P* = .827)
Racial fear	You	745	7	8 (*P* = .756)	7 (*P* = .975)	13 (*P* = .149)	4 (*P* = .343)
Violence	You or family member	657	13	10 (*P* = .306)	10 (*P* = .586)	13 (*P* = .988)	7 (*P* = .165)
Threatened or nonsexually harassed	You or family member	657	16	21 (*P* = .237)	23 (*P* = .243)	22 (*P* = .398)	18 (*P* = .854)
Sexual harassment	You or family member	657	9	8 (*P* = .706)	8 (*P* = .763)	6 (*P* = .526)	11 (*P* = .739)
*Actions based on concerns about discrimination*							
Avoided doctor or health care because of concerns of discrimination/poor treatment	You or family member	745	3	9 (*P* = .017)	14 (*P* = .039)	9 (*P* = .138)	5 (*P* = .488)
Avoided calling the police because of concerns of discrimination	You or family member	657	2	8 (*P* = .041)	8 (*P* = .118)	11 (*P* = .138)	5 (*P* = .457)

a Questions about you are personal experiences only; questions about you or family member ask if items have happened to you or a family member because you or they are [Asian OR white]; all adults asked about discrimination against [Asian Americans or whites] in America today.

b Individual questions only asked among a randomized subsample of half of respondents within each race category. Don't know/refused responses included in the total for unadjusted estimates.

c Non‐Hispanic white adults aged 18+.

d Non‐Hispanic Asian adults aged 18+.

e East Asians include adults who report being of Chinese, Japanese, Korean, and Taiwanese heritage.

f South Asians include adults who report being of Indian, Bangladeshi, Pakistani, and Sri Lankan heritage.

g Southeast Asians include adults who report being of Filipino, Vietnamese, Cambodian, Indonesian, Laotian, and Malaysian heritage.

h Question asked as “Generally speaking, do you believe there is or is not discrimination against [Asian Americans OR whites] in America today?”

i Equal pay question only asked among respondents who have ever been employed for pay.

j Jobs question only asked among respondents who have ever applied for a job.

k College application/attendance was only asked among respondents who have ever applied for college or attended college for any amount of time.

l Housing question only asked among respondents who have ever tried to rent a room or apartment, or to apply for a mortgage or buy a home.

m Question wording: “In your day‐to‐day life, have any of the following things ever happened to you [or a family member], or not?” and respondent indicated they had experienced this *and* believed this happened because you [or they] are [Asian American OR white]. Racial slurs = someone referred to you or a group you belong to using a slur or other negative word; microaggressions = someone made negative assumptions or insensitive or offensive comments about you; people acted afraid = people acted as if they were afraid of you.

n Significantly different from whites at *α* = 0.05 using the Holm‐Bonferroni method to sequentially correct for 80 comparisons between groups and models (shown in bold).

Table 2 also shows that 35 percent of Asian adults say they have experienced one or more types of interpersonal racial discrimination. More than a third (35 percent) of Asian American adults reported having personally been the target of racially based microaggressions, and 32 percent reported having been the target of racial slurs. One in five (21 percent) said they or a family member have been threatened or nonsexually harassed because they are Asian. Further, 10 percent said they or a family member have experienced violence because they are Asian, while 8 percent reported that they or a family member have experienced sexual harassment.

Anticipation or fear of experiencing discrimination also deterred some Asian adults from taking potentially needed action (Table 2). About one in ten (9 percent) Asian adults reported that, due to fear of discrimination or poor treatment, they have avoided the doctor or seeking health care for themselves or family, and 8 percent similarly said that they have avoided calling the police or other authority figures, even when in need.

### 3.3 Overall Asian‐white disparities in reported racial discrimination

Table 2 also presents uncontrolled comparisons of Asians and whites reporting these experiences of discrimination. Asian adults were more likely than whites to report having ever personally experienced racial discrimination in one or more institutional domains (37 percent vs 22 percent, *P* < .001), especially in housing (25 percent vs 5 percent, *P* < .001) (Table 2).

### 3.4 Asian heritage group‐white disparities in reported racial discrimination

Table 2 further shows that significant disparities exist between whites and Asian heritage groups, though these differences varied by domain. East Asians were significantly more likely than whites to report having ever experienced one or more forms of institutional discrimination (39 percent vs 22 percent, *P* < .001). South Asians were more likely than whites to report having ever experienced one or more forms of institutional discrimination (46 percent vs 22 percent, *P* < .001) and microaggressions (45 percent vs 19 percent, *P* < .001).

### 3.5 Adjusted odds of reporting personal experiences of racial discrimination

**Figure 1:**
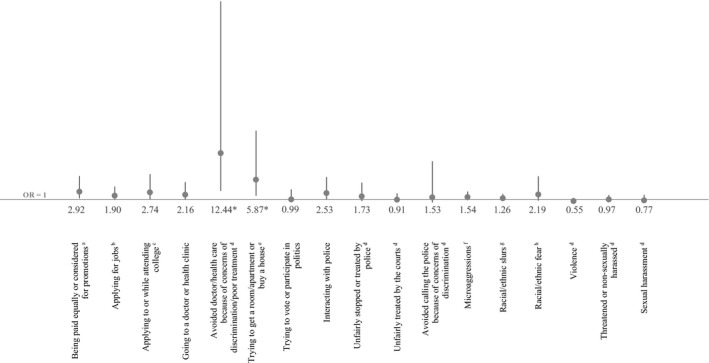
Adjusted odds of experiencing discrimination among Asians compared to whites (reference group). OR, odds ratio, with 95% confidence interval bars. Nationally representative sample of non‐Hispanic Asian and non‐Hispanic white adults aged 18+. *Indicates statistical significance at *α* < 0.05 using the Holm‐Bonferroni method to sequentially correct for 17 comparisons between models. Don't know/refused responses coded as missing. Odds ratios report the odds that Asians reported experiencing discrimination for each outcome (whites were the reference group). These estimates control for self‐ or interviewer‐reported gender of respondent, age (continuous), education (<college vs college graduate or more), household income (<$50 k or $50 k+), living in a neighborhood that is predominantly one's own race, household location (urban, suburban, rural), and region (Northeast, Midwest, South, West). Going to a doctor or health clinic and avoiding the doctor or health clinic also adjust for health insurance status (yes/no). ^a^Equal pay question only asked among respondents who have ever been employed for pay. ^b^Jobs question only asked among respondents who have ever applied for a job. ^c^College application/attendance was only asked among respondents who have ever applied for college or attended college for any amount of time. ^d^Includes discrimination against you or a family member. ^e^Housing question only asked among respondents who have ever tried to rent a room or apartment, or to apply for a mortgage or buy a home. ^f^Microaggressions indicate that someone made negative assumptions or insensitive or offensive comments about you because you are Asian American/white. ^g^Racial/ethnic slurs indicate that someone referred to you or your racial group using a slur or other negative word because you are Asian American/white. ^h^Racial/ethnic fear indicates that people acted as if they were afraid of you because you are Asian American/white

Figure 1 shows adjusted differences in the odds of Asian adults personally experiencing discrimination compared to whites (with full models shown in Appendices S2 and S3). The results show that, after accounting for potential social and demographic confounders in logistic regression models, there were Asian‐white disparities in reported discrimination in two domains: avoiding the doctor or health care and trying to rent or buy housing.

Table 3 presents adjusted odds of reporting discrimination among Asian Americans only. South Asians had higher odds of reporting discrimination in police interactions than East Asians (OR [95% CI] 4.03 [1.16, 14.02]), but this difference was not statistically significant. Asian women also had higher odds than Asian men of reporting discrimination when going to the doctor or health clinic (5.06 [1.13, 22.65]) and when trying secure housing (4.02 [1.13, 14.37]), but again, these differences did not achieve statistical significance.

**Table 3:** Odds of reporting personal experiences of racial discrimination across institutional domains among a sample of Asian adults in the United States

	Employment		Education	Health care		Housing	Political participation	Police and courts			
	Equal pay/promotions^b^	Applying for jobs^c^	College application/attendance^d^	Doctor or health clinic visits	Avoided doctor due to discrimination concerns	Trying to rent or buy a house^e^	Trying to vote or participate in politics	Interacting with police	Unfairly stopped or treated by the police	Unfairly treated by the courts	Avoided calling the police due to discrimination concerns
N^a^	149	150	152	182	183	145	158	164	171	—	168
**OR (95% CI)**											
Gender^f^											
Male	Ref	Ref	Ref	Ref	Ref	Ref	Ref	Ref	Ref	—	Ref
Female	1.31 (0.46, 3.78)	1.56 (0.60, 4.07)	1.55 (0.55, 4.33)	5.06 (1.13, 22.65)	0.95 (0.26, 3.55)	4.02 (1.13, 14.37)	0.55 (0.13, 2.26)	2.60 (0.82, 8.26)	2.21 (0.69, 7.04)	—	2.11 (0.43, 10.42)
Age	1.02 (0.99, 1.05)	1.00 (0.97, 1.03)	0.96 (0.93, 0.99)	1.00 (0.97, 1.03)	1.02 (0.98, 1.06)	1.02 (0.99, 1.06)	1.00 (0.96, 1.04)	0.98 (0.95, 1.00)	0.96 (0.92, 1.00)	—	0.99 (0.94, 1.04)
Education											
<College	Ref	Ref	Ref	—	—	Ref	Ref	Ref	Ref	—	Ref
College+	1.35 (0.41, 4.51)	1.05 (0.38, 2.91)	0.91 (0.28, 3.06)	—	—	2.96 (0.66, 11.22)	4.59 (1.18, 17.85)	1.84 (0.39, 8.63)	1.29 (0.30, 5.60)	—	0.91 (0.23, 3.62)
Income											
<$50 k	Ref	Ref	Ref	Ref	Ref	Ref	Ref	Ref	Ref	—	Ref
≥$50 k	2.91 (0.81, 10.52)	0.56 (0.19, 1.62)	2.86 (0.62, 13.27)	0.84 (0.14, 5.06)	0.57 (0.11, 2.94)	0.74 (0.20, 2.77)	0.15 (0.03, 0.74)	2.41 (0.53, 10.94)	1.73 (0.39, 7.69)	—	0.34 (0.05, 2.17)
Geographic heritage group^g^											
East Asian^h^	Ref	Ref	Ref	Ref	Ref	Ref	Ref	Ref	Ref	—	Ref
South Asian^i^	1.26 (0.49, 3.20)	1.39 (0.52, 3.71)	0.48 (0.17, 1.40)	4.45 (0.63, 31.44)	0.95 (0.22, 4.16)	2.12 (0.52, 8.71)	1.92 (0.48, 7.64)	1.00 (0.34, 2.89)	4.03 (1.16, 14.02)	—	2.00 (0.54, 7.46)

Note

Nationally representative sample of Asian adults aged 18+. Responses to questions of unfair treatment by the court system could not be modeled due to small sample size and the rarity of affirmative responses to this question. Education could not be added as a covariate to models of discrimination in healthcare settings due to the rarity of affirmative responses to this question among those with less than a four‐year college degree.

Abbreviations: CI, confidence interval; OR, odds ratio.

a Individual questions only asked among a randomized subsample of half of respondents. Don't know/refused responses coded as missing.

b Equal pay question only asked among respondents who have ever been employed for pay.

c Jobs question only asked among respondents who have ever applied for a job.

d College application/attendance was only asked among respondents who have ever applied for college or attended college for any amount of time.

e Housing question only asked among respondents who have ever tried to rent a room or apartment, or to apply for a mortgage or buy a home.

f Gender indicates self‐ or interviewer‐reported gender of respondent.

g Southeast Asians were omitted from this analysis due to sample size limitations. Results here reflect comparisons between East and South Asians only.

h East Asians include adults who report being of Chinese, Japanese, Korean, or Taiwanese heritage.

i South Asians include adults who report being of Indian, Bangladeshi, Pakistani, or Sri Lankan heritage.

Even though the lower bound confidence intervals for some estimates are above 1, after using the Holm‐Bonferroni method to sequentially correct for 48 comparisons between groups and models, no estimates in this model remained statistically significant at α<0.05.

Age and education also showed some associations with discrimination; however, these differences were not statistically significant. Increasing age was associated with lower odds of reporting discrimination in college applications/attendance. Compared to having some college or less, having at least a college degree was associated with higher odds of discrimination in political participation.

The results displayed in Figure 1, Table 3, and Appendices S2 and S3 remained mostly robust to alternate model specifications. The Holm‐Bonferroni correction we used, however, meant that slight changes in p‐values at the thousandth or ten‐thousandth level could change a model's statistical significance. This happened in our model of adjusted differences in the odds of Asian adults personally experiencing discrimination when buying or renting housing, compared to whites (Figure 1, Appendices S2 and S3). When we substituted a dichotomous age variable split at 40 years for our continuous age variable, when we respecified our education variable to capture whether someone had a postgraduate degree or less, and when we dropped all but one or two covariates in the model, changes in p‐values at the thousandth or ten‐thousandth level changed the statistical significance of the model. In Table 3, adding foreign birthplace to our model and substituting it for Asian heritage groups, where sample size allowed, did not change any of our findings. Our results in Table 3 also did not change when we substituted alternate specifications for our age, income, and education variables.

## 4 DISCUSSION

Three key findings emerged from this national survey of Asian American adults. First, our results show that Asians in the United States experience discrimination across many areas of life, particularly in interpersonal interactions, and also in institutional domains such as housing and health care. As more than one in seven Asian adults reported discrimination in clinical encounters, our findings on healthcare discrimination add to a growing body of studies that document patients' continued perceptions of unfair treatment in health care,^37^, ^38^, ^39^ which affect Asian Americans' avoidance of care^26^, ^27^ and their physical and mental health.^11^, ^12^, ^13^, ^14^, ^15^, ^16^

Second, our findings indicate that, even after adjusting for major sociodemographic characteristics, Asian Americans have significantly higher odds than whites of avoiding the doctor due to fear of racial discrimination, and also reporting racial discrimination in housing. Not only do these findings violate a societal expectation of equal treatment and racial equality, they also raise health equity concerns, given the association between discrimination and myriad negative health outcomes.^11^, ^12^, ^13^, ^14^ The demonstrated association between discrimination against Asians and serious health problems documented in other research, including major depression, anxiety, suicidal ideation, cardiovascular disease, respiratory conditions, pain disorders, and worse physical functioning, suggests that the high prevalence of racial discrimination across domains documented in this study should be interpreted as a threat to the physical and mental well‐being of Asians in America.^11^, ^12^, ^16^, ^22^

Third, our findings suggest that some Asians may face more discrimination than others. We found that Asian women had higher odds of experiencing discrimination when seeking health care and when trying to obtain housing, compared to Asian men. While these results were not statistically significant after using the Holm‐Bonferroni correction, we believe it is important to conduct future research on gender differences with larger sample sizes, given previous evidence indicating that experiences of discrimination in healthcare settings among Asian women may disproportionately decrease their use of formal healthcare services.^10^ Furthermore, our analyses indicate South Asians may be more likely than both whites and East Asians to report racial discrimination in institutional and interpersonal settings. Even though these differences were also not statistically significant after adjusting for multiple comparisons, given the results from our bivariate analyses, future research should explore whether South Asian women may be more likely to experience a unique form of compounded discrimination that could put them at greater risk for concomitant negative physical and mental health outcomes.

We believe this study represents an important contribution to the literature because few studies have examined experiences of discrimination at the population level and fewer still report the experiences of disaggregated groups of Asian Americans.^11^ In fact, most studies comparing discrimination and health between South Asians and other groups have occurred in the United Kingdom, rather than the United States.^18^, ^40^ Our results indicate that studies treating Asian Americans as a homogenous group should be read with caution. Future research should strive to disaggregate Asians by ethnicity, heritage, and/or gender (at a minimum) in order to examine the unique experiences and vulnerabilities of Asian subgroups. Future studies should also investigate whether Asian Americans experience discrimination in additional domains (eg, obtaining health insurance, getting loans or mortgages), as well as how discrimination, especially in nonhealthcare settings, affects physical and mental health outcomes. Furthermore, policymakers, as well as health and housing administrators, may need to account for intragroup differences as they consider interventions that target those at risk of experiencing discrimination.

### 4.1 Limitations

Our results should be interpreted considering several limitations. Due to the cross‐sectional design of this study, we cannot determine the timing or severity of experiences of discrimination. Although interviews were offered in Asian languages, some respondents may have nevertheless been excluded from the study due to language barriers. Respondents may have also interpreted questions differently based on varying backgrounds and expectations, and they may have reported experiences of cultural or communication problems as discrimination. Due to sample size, this survey grouped respondents into three large heritage groups, rather than individual ethnic identities. While better than aggregate data, which often treat Asians as a monolithic group, this approach still obscures a multitude of linguistic, cultural, and religious differences, all of which may impact experiences of discrimination and health. Sample size also limited our ability to estimate complex models and test any potential interaction effects. Our low response rate is an additional limitation, though evidence suggests that low response rates do not bias results if the survey sample is representative of the study population.^30^, ^31^ Recent research has shown that such surveys, when based on probability samples and weighted using US Census parameters, yield accurate estimates in most cases when compared with both objective measures and higher response surveys.^30^, ^31^ For instance, a recent study showed that across fourteen different demographic and personal characteristics, the average difference between government estimates from high‐response rate surveys and a Pew Research Center poll with a response rate similar to this poll was 3 percentage points.^30^, ^41^, ^42^ However, it is still possible that some selection bias may remain that is related to the experiences being measured. Despite these limitations, this study design allowed us to closely examine reported experiences of racial discrimination among Asian adults. Our results highlight the extent of discrimination currently experienced by Asian Americans across a variety of domains and interpersonal interactions, which may carry severe economic, social, and health consequences.

## 5 CONCLUSIONS

Asians in the United States experience discrimination interpersonally and across many institutional settings, such as housing and health care. The threat of racial discrimination and poor treatment also makes Asian Americans more likely than their white counterparts to avoid going to the doctor or seeking health care. Among Asian Americans, South Asians may be especially vulnerable to institutional and interpersonal forms of discrimination, including microaggressions. Our findings add to a growing body of literature on the unique needs of Asian communities and subpopulations that may be particularly vulnerable to structural and relational forms of discrimination. These findings have major implications for healthcare providers, researchers, policymakers, employers, and government agencies that interact with and impact Asian American communities.
